# Tislelizumab Combined With Axitinib in Neoadjuvant Treatment of Locally Advanced Clear Cell Renal Cell Carcinoma: A Single‐Center, Phase II Clinical Study

**DOI:** 10.1002/mco2.70641

**Published:** 2026-02-17

**Authors:** Wenjin Yang, Shun Zhang, Guangxiang Liu, Hao Li, Xin Wang, Bo Jiang, Gutian Zhang, Hongqian Guo, Changwei Ji

**Affiliations:** ^1^ Department of Urology Nanjing Drum Tower Hospital, Affiliated Hospital of Medical School, Nanjing University Nanjing jiangsu China; ^2^ Institute of Urology Nanjing University Nanjing jiangsu China; ^3^ Department of Urology Nanjing Drum Tower Hospital, Clinical College of Nanjing Medical University Nanjing jiangsu China

**Keywords:** advanced clear cell renal cell carcinoma, immunotherapy, neoadjuvant treatment, objective response rate, targeted therapy

## Abstract

Clear cell renal cell carcinoma (ccRCC) accounts for 70%‒80% of renal cell carcinoma cases and often shows no symptoms in early stages. Thus, approximately 30% of patients are diagnosed with advanced ccRCC. This single‐center prospective single‐arm study evaluated the efficacy and safety of tislelizumab combined with axitinib in patients with locally advanced ccRCC. A total of 20 eligible patients were enrolled at Nanjing Drum Tower Hospital from September 2021 to June 2024. The primary endpoint was objective response rate (ORR) before surgery, and secondary endpoints included disease‐free survival (DFS), overall survival, safety, and tissue biomarker analysis. All patients completed neoadjuvant treatment, and 19 underwent planned surgery; 70% (14/20) had cT3 stage disease with a median tumor diameter of 8.3 cm. The ORR was 55% (11 partial responses), 73.6% (14/19) achieved pathological downstaging, one patient attained pathological complete response, and no grade ≥3 perioperative complications occurred. The 2‐year DFS rate was approximately 90%, and biomarker analysis showed significantly higher tumor shrinkage rates in patients with RTK/RAS pathway alterations. In conclusion, tislelizumab combined with axitinib exhibits substantial efficacy and acceptable safety in neoadjuvant treatment of locally advanced ccRCC, providing preliminary clinical evidence for its application.

## Graphical abstract

Tislelizumab plus axitinib as neoadjuvant therapy for locally advanced clear cell renal cell carcinoma (ccRCC) demonstrated substantial efficacy and acceptable safety in a single‐center prospective single‐arm study. The objective response rate reached 55%, one patient attained pathological complete response, and patients with RTK/RAS pathway alterations revealed significantly higher tumor shrinkage, providing preliminary clinical evidence for the regimen application in locally advanced ccRCC.

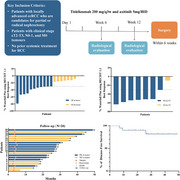

## 1 Introduction

Clear cell renal cell carcinoma (ccRCC) is the most common form of renal cell carcinoma (RCC), accounting for approximately 70%‒80% of all RCC cases [1, 2]. While modern diagnostic technology continues to improve, enabling the early detection of small renal tumors, approximately 30% of patients are still diagnosed with locally advanced or metastatic RCC at the time of their initial diagnosis [3]. Locally advanced ccRCC is highly invasive and tends to metastasize. Consequently, early treatment strategies primarily depend on surgical resection of the tumor [4].

However, as the disease progresses, surgery alone often cannot meet the treatment needs of patients with locally advanced ccRCC. Researchers are exploring new treatment strategies to improve both survival rates and quality of life for patients with locally advanced ccRCC. Despite advancements in treatment modalities, the effectiveness of existing therapies remains limited, especially in patients with locally advanced or metastatic disease. In recent years, targeted combined immunotherapy has emerged as a promising approach that may enhance surgical resection and pathological response rates for a range of tumor types [5, 6]. This therapy offers a groundbreaking strategy that could significantly enhance the treatment of locally advanced ccRCC.

Studies have shown that immune checkpoint inhibitors (ICIs) enhance the attack on tumors by activating the host immune system, while targeted therapies work by directly inhibiting the growth and spread of tumor cells [7]. For example, the combination of pembrolizumab and axitinib improved survival rate compared with that in sunitinib monotherapy in patients with metastatic renal cell carcinoma [8]. While neoadjuvant therapy demonstrates significant promise, it faces several challenges. One major issue is the development of drug resistance after treatment, which results in reduced efficacy [9].

Despite existing studies, there is a significant gap in systematic research on locally advanced ccRCC, particularly in evaluating the efficacy of neoadjuvant therapy that includes tislelizumab and axitinib. Developing a neoadjuvant regimen that promotes tumor shrinkage and immune activation is crucial for patients with locally advanced ccRCC.

This study adopted a single‐center phase II clinical design to assess the efficacy of a neoadjuvant treatment regimen combining tislelizumab and axitinib, in patients with locally advanced ccRCC. The primary goal was to reduce the tumor size for surgical resection while ensuring the best possible nephron preservation, preventing recurrence, and promoting long‐term survival. This study uniquely contributes to the field by evaluating the real‐world effects and safety of this combination treatment, addressing existing gaps in the literature, and providing essential data for future large‐scale multicenter studies. In addition, the study design allows for a comprehensive analysis of biomarkers, treatment responses, and clinical characteristics in patients, aiming to uncover factors that affect treatment outcomes.

## 2 Results

Between September 2021 and June 2024, 20 eligible patients were enrolled. All of them completed neoadjuvant treatment with tislelizumab and axitinib. A total of 19 patients underwent surgery as planned. Among them, 70% (14/20) had a clinical stage of cT3. The median tumor diameter at baseline was 8.3 cm (range: 4.8‒14.4 cm). Furthermore, 40% (8/20) had tumor thrombi. Detailed baseline characteristics of the study population are presented in Table 1.

**TABLE 1:** Patient baseline characteristic.

Variable	*N* = 20
Age at time of inclusion (range) (years)	66 (29‒78)
Sex, *n* (%)	
Male	10 (50%)
Female	10 (50%)
ECOG performance status, *n* (%)	
0	17 (85%)
1	3 (15%)
BMI	22.8 (18.9‒31.9)
Primary tumor stage, *n* (%)	
cT2a	4 (20%)
cT2b	2 (10%)
cT3a	7 (35%)
cT3b	6 (30%)
cT3c	1 (5%)
Regional lymph node stage, *n* (%)	
N1	2 (10%)
Side	
Left	9 (45%)
Right	10 (50%)
Bilateral	1 (5%)
Baseline tumor diameter (range) (cm)	8.3 (4.8‒14.4)
With tumor thrombus, n (%)	8 (40%)

**Abbreviation:** BMI, body mass index.

### 2.1 Radiological Efficacy

**FIGURE 1:**
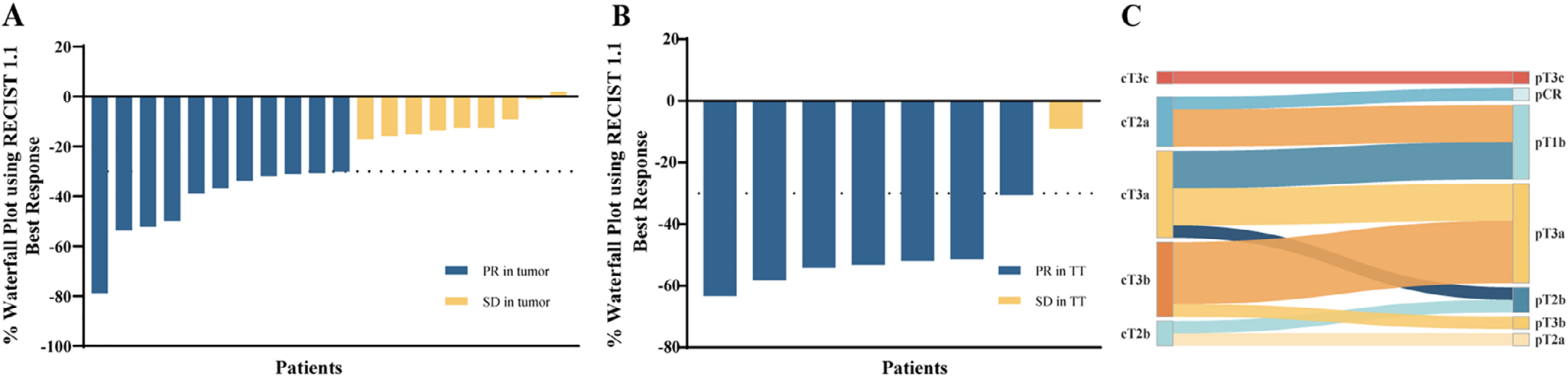
Treatment response. (A) Imaging assessment of primary tumor response. (B) Imaging assessment of tumor thrombus response. (C) Differences in imaging and pathological staging of patients before and after treatment.

There was a statistically significant difference in the median maximum tumor diameter before and after treatment: a decrease from 8.3 cm ± 4.1 cm to 5.6 cm ± 3.3 cm (*p* < 0.001). A total of 19 patients showed a decrease in the maximum diameter of the primary tumor, with a median reduction of 30.2% (range: −1.8% to 78.9%). Further analysis demonstrated that 11 out of 20 patients achieved a partial response (PR), while another nine exhibited stable disease (SD), which resulted in an objective tumor response rate (ORR, the percentage of people in a study or treatment group who have a partial response or complete response to the treatment within a certain period of time) of 55% (Figure 1A). Among the eight patients with tumor thrombi, there was a statistically significant difference in the median length of the tumor thrombi before and after treatment: 4.7 cm ± 3.5 cm to 2.3 cm ± 2.1 cm (*p* = 0.016). The median reduction in the length of tumor thrombus was 53.2% (range: 9.1%‒63.3%) (Figure 1B).

A total of 14 (70%) patients achieved downstaging after treatment. Nine patients (45%) had their treatment regimens changed due to neoadjuvant therapy. Among them, two patients whose tumors were initially considered inoperable achieved tumor resection after treatment. Seven patients who were originally scheduled for radical nephrectomy (RN) underwent partial nephrectomy (PN) after treatment. Although, in one patient the tumor grew larger, no disease progression was detected in any of the patients during preoperative drug treatment.

### 2.2 Perioperative Treatment

Among the 20 patients, 19 underwent a surgical procedure. Two patients underwent extensive open surgery. After the surgery, they were transferred to the intensive care unit for 1 day. The following day, after their condition stabilized, they returned to the department of urology to continue treatment. There were no perioperative deaths. According to the Clavien‒Dindo grading system, no perioperative complications of grade III or higher were observed. The detailed data are presented in Table 2.

**TABLE 2:** Perioperative data.

Variable	*N* = 19
Type of surgery, *n* (%)	
Open	3 (15.8%)
Robotic RN	7 (36.8%)
Robotic PN	7 (36.8%)
Laparoscopic RN	2 (10.6%)
Degree of tissue adhesion, *n* (%)	
Mild/none	10 (52.6%)
Moderate	3 (15.8%)
Severe	6 (31.6%)
Surgical time (range) (min)	215 (90‒310)
Intraoperative blood loss (range) (mL)	100 (20‒1600)
Postoperative ICU management	
Number, *n* (%)	2 (10.5%)
Time (days)	1
Postoperative drainage tube removal time (range) (days)	3 (1‒10)
Postoperative hospital stay (range) (days)	4 (2‒14)
Postoperative complications, *n* (%)	
Grade I	7 (36.8%)
Grade II	4 (21.1%)
Grade III	0
Grade IV	0
Grade V	0

Abbreviation: ICU, intensive care unit.

### 2.3 Pathological Response

Among the 19 patients evaluated, 14 (73.6%) experienced a decrease in their pathological stage following treatment. This included three patients whose status was downgraded from clinical stage cT3 to pathological stage pT1, and one patient who achieved pathological complete response (pT0) (Figure 1C).

### 2.4 Drug Safety

During treatment, the most common adverse event reported by patients were hematological toxicity (eight cases, 40%), abnormal thyroid function (five cases, 25%), nausea and vomiting (three cases, 15%), and loss of appetite (two cases, 10%). Adverse events related to treatment that were graded IV or V. Three patients stopped taking the drug for one cycle due to severe adverse reactions and were subjected to surgery earlier than scheduled. The remaining 16 patients completed their drug treatment as planned and then underwent surgery.

### 2.5 Follow‐Up

**FIGURE 2:**
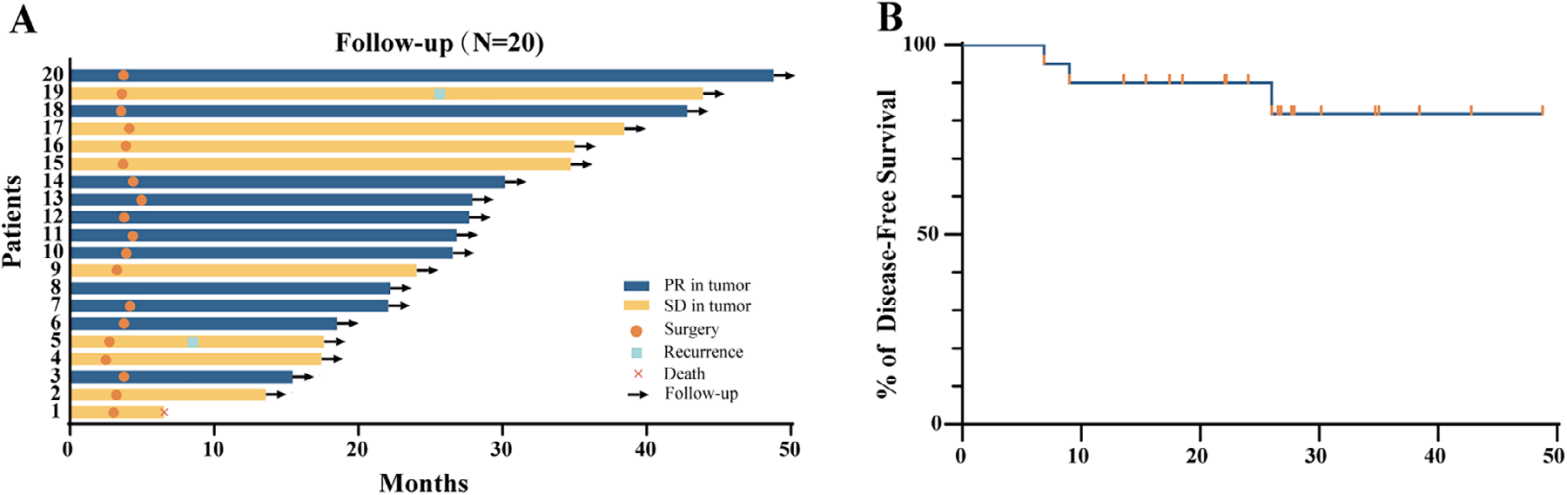
Follow‐up data. (A) Overall survival distribution of patients. (B) Disease‐free survival analysis curve.

The median follow‐up time was 26.3 months (range: 6.5‒48.8 months). During this follow‐up, two patients had disease recurrence, and one patient died because of COVID‐19 (Figure 2A). The disease‐free survival (DFS, the time between starting treatment aimed at curing cancer, and signs that the cancer has relapsed) rate at 2 years was approximately 90% (95% CI: 65.6%‒97.4%) (Figure 2B). After surgery, only one patient who underwent open surgery had abnormal long‐term creatinine levels, while the creatinine levels of the other patients remained stable.

### 2.6 Biomarkers

**FIGURE 3:**
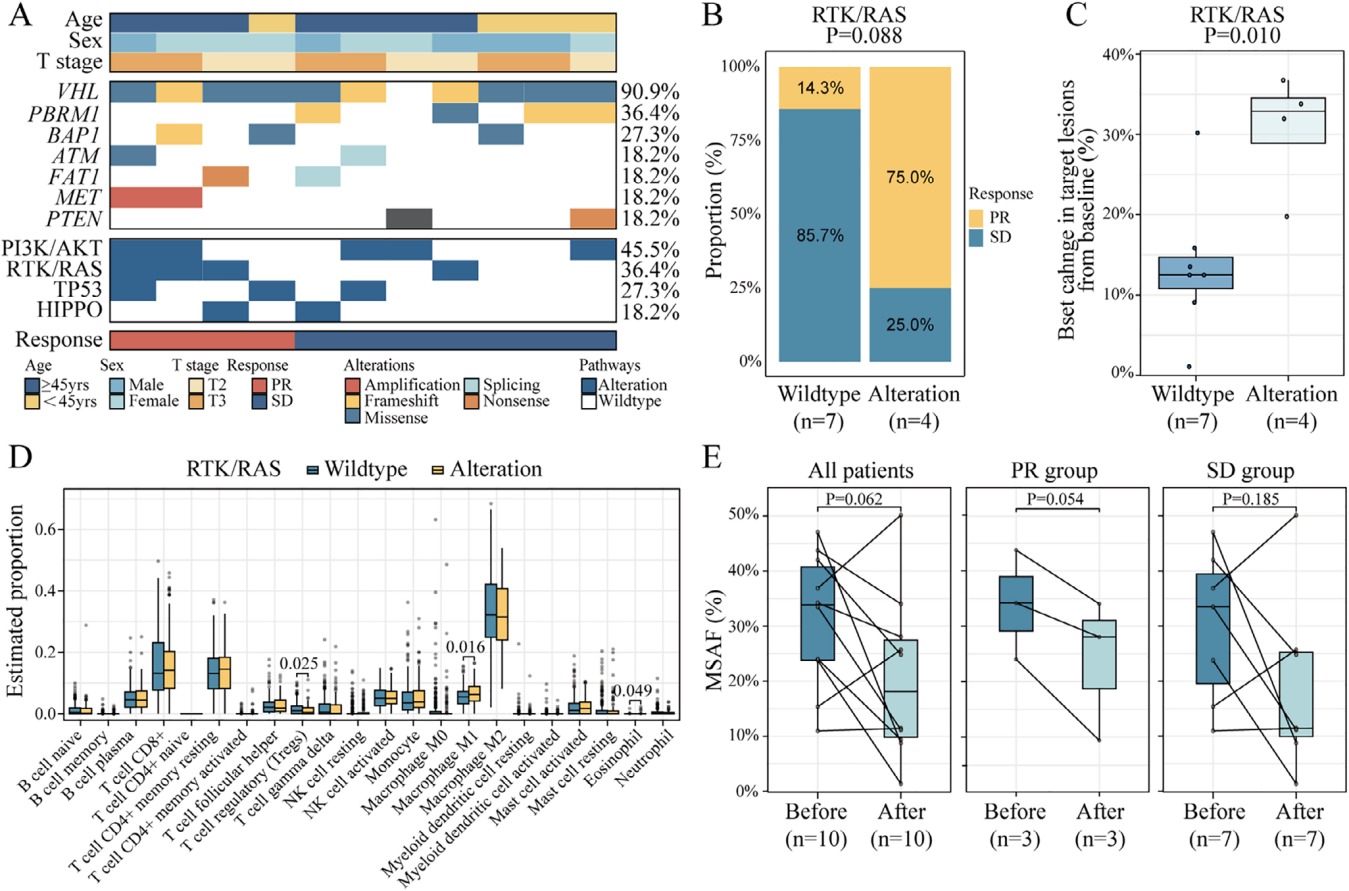
Biomarker analysis of RTK/RAS pathway alterations and response to ICI treatment in 11 ccRCC patients. (A) The genomic landscape of 11 ccRCC patients. (B) Differences in PR rates to ICI treatment in patients with RTK/RAS pathway alterations. (C) Tumor shrinkage rates in response to ICI treatment in ccRCC patients stratified by RTK/RAS pathway alteration status. (D) Infiltration score of macrophages in the RTK/RAS pathway alteration group. (E) Changes in tissue mutation abundance before and after treatment.

NGS was performed on 13 ccRCC patients who provided sufficient pre‐treatment biopsy samples, and two patients were omitted from the analysis due to poor quality control. The genomic landscape of 11 ccRCC patients is shown in Figure 3A. The most frequently mutated genes were VHL (90.9%), PBRM1 (36.4%), and BAP1 (27.3%). Approximately 45.5% of patients with ccRCC harbored PI3K‐AKT‐related gene alterations. Alterations in other cancer‐related pathways were also observed in patients with ccRCC, including in the receptor tyrosine kinase (RTK)/small GTPase (RAS) (*n* = 4, 36.4%), TP53 (*n* = 3, 27.3%), and HIPPO (*n* = 2, 18.2%) pathways. No specific gene variants were significantly associated with treatment response (Table S1). Notably, patients with altered RTK/RAS pathway had a higher PR rate (75.0% vs. 25.0%; *p* = 0.088) and significantly greater tumor shrinkage (median: 32.9% vs. 12.5%; *p* = 0.010) in response to ICI treatments compared with that in wild‐type RTK/RAS pathway (Figure 3B,C). To explore the underlying mechanisms of the predictive role of RTK/RAS pathway in ICI efficacy, we obtained RNA‐seq data for ccRCC patients from the TCGA‐KIRC cohort and estimated the abundance of immune cells. Infiltration scores for macrophage M1 (*p* = 0.016) and eosinophil (*p* = 0.049) were significantly elevated in the RTK/RAS pathway‐altered group, whereas regulatory T‐cell (*p* = 0.025) infiltration scores were significantly decreased (Figure 3D). These results suggest that the sensitivity to immunotherapy of RTK/RAS pathway‐altered patients may be attributed to the activation of the immune microenvironment. Next, we assessed the tumor mutational burden of pre‐treatment biopsy samples. There were no significant differences in the mutational burden between the PR and SD patients (*p* = 0.763; Figure S1). Moreover, pre‐ and post‐treatment paired samples were analyzed in 10 patients. The results showed that the Maximum Somatic Mutation Allele Fraction tends to be lower in the post‐treatment samples (*p* = 0.062), particularly in the PR group (*p* = 0.054; Figure 3E).

## 3 Discussion

This study adopted a single‐center, phase II clinical study design to evaluate the efficacy and safety of neoadjuvant therapy with tislelizumab combined with axitinib in patients with locally advanced ccRCC. The results showed that our neoadjuvant therapy exhibited positive effects in terms of tumor shrinkage rate, surgical resection rate, and survival, and was well tolerated by patients.

After 12 weeks of treatment with axitinib combined with tislelizumab, 19 patients (95%) showed a reduction in the maximum diameter of the primary tumor, with a median reduction rate of 30.2% (range: −1.8% to 78.9%) and an ORR of 55%. Compared with previous studies using single‐agent or combined neoadjuvant therapy, our regimen demonstrated an advantage in terms of tumor shrinkage efficiency. In a randomized trial using pazopanib or sunitinib, 76.9% of patients showed tumor shrinkage, with a median reduction rate of 20.5% in the maximum tumor diameter and a maximum reduction rate of 60% [10]. A study using the ICI nivolumab alone showed that the median change rate of the primary tumor maximum diameter was only +0.85% (range: −6.2% to +7.9%) [11]. In terms of neoadjuvant therapy with combined targeted and immune therapies, an early study of the NEOAVAX regimen using 12 weeks of neoadjuvant avelumab combined with axitinib showed a median tumor reduction of 20% (range: 0%‒43.5%) and an ORR of 30% [12]. Another 8‐week phase II clinical study of sitravatinib combined with nivolumab (NCT03680521) reported a median tumor reduction rate of 13.5% (range: 0%‒33%) and an ORR of 11.8% [13]. A recently published phase II study of toripalimab combined with axitinib showed a median tumor reduction of 26.7% (range: −2.0% to 40.3%) and an ORR of 45%, with a 2‐year estimated DFS similar to our results [14]. Except for the last study, the tumor reduction efficiency and ORR of other trials were less than optimal. This further proves the superiority of our regimen.

Since Labbate et al. first reported complete remission of extensive vena cava tumor thrombi with neoadjuvant immunotherapy [15], research on neoadjuvant therapy for RCC with venous tumor thrombi has gradually deepened. Candelario et al. used axitinib and pembrolizumab to treat five patients with RCC with tumor thrombi, of whom four responded well to the treatment and the length of the tumor thrombi was reduced [16]. In addition, in the NAXIVA study, a phase II study of axitinib alone showed that the length of venous tumor thrombi in 75% (15/20) of patients was reduced [17]. The results of the recent NEOTAX study showed that in a phase II clinical trial of toripalimab combined with axitinib, 44.0% (11/25) of patients had a decrease in thrombus levels [18]. In our study, eight patients with tumor thrombi showed a trend toward thrombus shrinkage after treatment, and surgery was successfully completed with negative resection margins. These findings suggest that neoadjuvant treatment with axitinib combined with tislelizumab effectively reduces venous tumor thrombi and provides more favorable conditions for subsequent surgery, improving the feasibility and safety of surgery.

Regarding nephrectomy outcomes, seven patients (35%) required RN before treatment, but after neoadjuvant therapy, tumor size was reduced sufficiently to allow PN. The kidney preservation rate in our study was lower than reports of treatment with axitinib alone [19, 20], this may be partly due to the inclusion of patients with cT3b and cT3c tumors or venous tumor thrombus, which limits the feasibility of PN. Importantly, our robot‐assisted laparoscopic PN achieved a 100% negative surgical margin rate on pathological examination. Notably, two of these seven patients had a solitary kidney (one had the contralateral kidney removed due to renal cancer 10 years prior, and the other had a unilateral resection because the contralateral kidney was found to be non‐functional 6 years prior). Neoadjuvant therapy allowed preservation of their only remaining kidney, successfully maintaining and avoid long‐term dialysis.

In terms of drug safety, our results are consistent with previous studies on neoadjuvant therapy, with the most common treatment‐related adverse events including hematological toxicity, gastrointestinal reactions, and thyroid dysfunction [21, 22, 23]. Adverse events affected the course of treatment in three patients, resulting in early surgery. Specifically, one patient suspended the fourth treatment cycle due to severe nausea, vomiting, and sudden high fever at the end of the third cycle and underwent surgery immediately after evaluation. The second patient was diagnosed with organic heart lesions during the third cycle of treatment (unrelated to drug treatment), resulting in early termination of neoadjuvant therapy and surgery. The third patient terminated drug treatment in the first week of the fourth cycle due to severe liver damage and hypoproteinemia, followed by early surgery. No grade IV or V adverse events were observed in our study. These events are influenced by drug characteristics, administration methods, and individual patient differences. In future studies, these mechanisms should be explored in depth to improve patient safety and treatment experience. Following neoadjuvant therapy, no patients experienced perioperative complications of grade III or higher, which indicates certain advantages compared with some earlier studies. This outcome not only reflects the maturity of surgical techniques, but also demonstrates the perioperative safety of axitinib combined with tislelizumab neoadjuvant therapy.

Currently, existing studies on neoadjuvant therapy paid little attention to the perioperative and long‐term renal function of patients; however, we specifically focused on this aspect. Patients with locally advanced ccRCC often present with poor renal function, yet in our study, patients’ renal function remains generally stable after surgery in those who receive neoadjuvant therapy, and the drugs do not appear to have a significant impact on renal function. This finding represents an important advantage for the long‐term quality of life of patients after surgery.

In recent years, with the development of molecular biology and genomics, the role of biomarkers in ccRCC has received increasing attention. These biomarkers can help identify the tumor characteristics and predict the patient's response to different treatment options, thereby providing a basis for personalized treatment. For example, vascular endothelial growth factor (VEGF) and its related signaling pathways play an important role in the occurrence and development of ccRCC and have become one of the main targets of targeted therapy [24]. Changes in the DNA damage response pathway in tumor cells are also considered closely related to the prognosis of locally advanced ccRCC, and mutations in the DNA damage response pathway are significantly associated with the DFS of the patient [25]. The infiltration of immune cells in the tumor microenvironment is closely related to the patient's prognosis, while specific immune cell subsets (such as CD8^+^ T cells) are associated with the tumor's immune escape mechanism [26].

In our study, we found that compared with the wild‐type RTK/RAS pathway, patients with altered RTK/RAS pathways had a significantly increased tumor shrinkage rate (*p* = 0.010), which was not reported in previous biomarker analyses of neoadjuvant therapy studies. Some basic studies have shown that abnormal activation of RTK and RAS signaling pathways in renal cancer cells is closely related to the occurrence and progression of renal cancer. RTK activates downstream signal transduction pathways by binding to its ligand, thereby affecting biological behaviors such as cell proliferation, survival, and migration [27, 28]. In patients with renal cancer, overexpression of RTK is often associated with tumor malignancy, poor prognosis, and drug resistance. Overexpression of specific RTKs such as VEGFR and platelet‐derived growth factor receptor is closely related to the high invasiveness and metastatic ability of renal cancer [29]. Activation of VEGFR promotes angiogenesis in the tumor microenvironment, allowing tumor cells to obtain more oxygen and nutrients, thereby accelerating tumor growth and metastasis. Currently, targeted therapeutic strategies for the RTK/RAS pathway are being actively explored. For example, drugs targeting RAS mutations have entered the clinical trial stage. These drugs aim to improve patient prognosis by inhibiting this pathway [30]. Furthermore, studies have shown that the combined use of RTK/RAS pathway inhibitors with other treatments (such as chemotherapy or immunotherapy) may enhance therapeutic effectiveness [31]. Our findings reveal that the RTK/RAS pathway significantly increases the tumor shrinkage rate of patients, which may provide new theoretical basis and clinical guidance for renal cancer diagnosis and treatment, and serve as a reference for the study of other types of tumors.

Future research should focus on revealing the specific mechanism of action of the RTK/RAS pathway in‐depth and its unique manifestations in different subtypes of renal cancer. This will help to understand the heterogeneity of renal cancer and provide a theoretical basis for the development of new treatment strategies. In addition, the integrated analysis of genomics and clinical data will help identify new biomarkers, thereby improving the accuracy of personalized treatment.

Although this study presents several innovations and new findings, it has certain limitations. These primarily include a small sample size, the absence of a control group, and insufficient long‐term follow‐up data. The small sample size may reduce the statistical power of the results, limiting their generalizability. In addition, as this study adopts a single‐center design, selection bias may be present, restricting external validation of the results. The lack of a control group prevents direct comparison of the efficacy of tislelizumab combined with axitinib with other treatment options, which limits the clinical applicability of our findings. Furthermore, the lack of long‐term follow‐up data hinders a complete assessment of the durability of efficacy and safety. Therefore, future studies should consider expanding the sample size, incorporating a control group, and conducting long‐term follow‐up to evaluate the efficacy and safety of this treatment regimen more comprehensively.

## 4 Conclusion

This study showed that tislelizumab combined with axitinib has good efficacy and safety in neoadjuvant treatment of patients with locally advanced ccRCC. Our findings provide preliminary evidence for the clinical application of this treatment strategy.

## 5 Materials and Methods

### 5.1 Experimental Design

This single‐center, single‐arm phase II clinical trial aims to evaluate the ORR and safety of combining neoadjuvant immunotherapy with targeted therapy in T2‐T3N0‐N1M0 ccRCC patients.

### 5.2 Experimental Subjects

Inclusion criteria: in this study, patients aged 18 years or older diagnosed with ccRCC at stages T2 to T3 and N0 to N1 were confirmed by pathological biopsy. After completing the initial neoadjuvant therapy, patients underwent RN or PN when necessary. Other criteria were: an Eastern Cooperative Oncology Group score of 0‒1; normal blood cell production; normal organ function; and the ability to understand and follow the planned treatment, laboratory tests, and research procedures.

Exclusion criteria: patients with other malignant tumors that have progressed or advanced in the past 5 years; patients who have received anti‐tumor treatments, including immunotherapy, targeted therapy, or cytokine therapy; and patients with diseases or medications that could impact drug evaluation, such as severe cardiovascular or cerebrovascular diseases, or mental health conditions.

### 5.3 Treatment Plan and Follow‐Up

**FIGURE 4:**
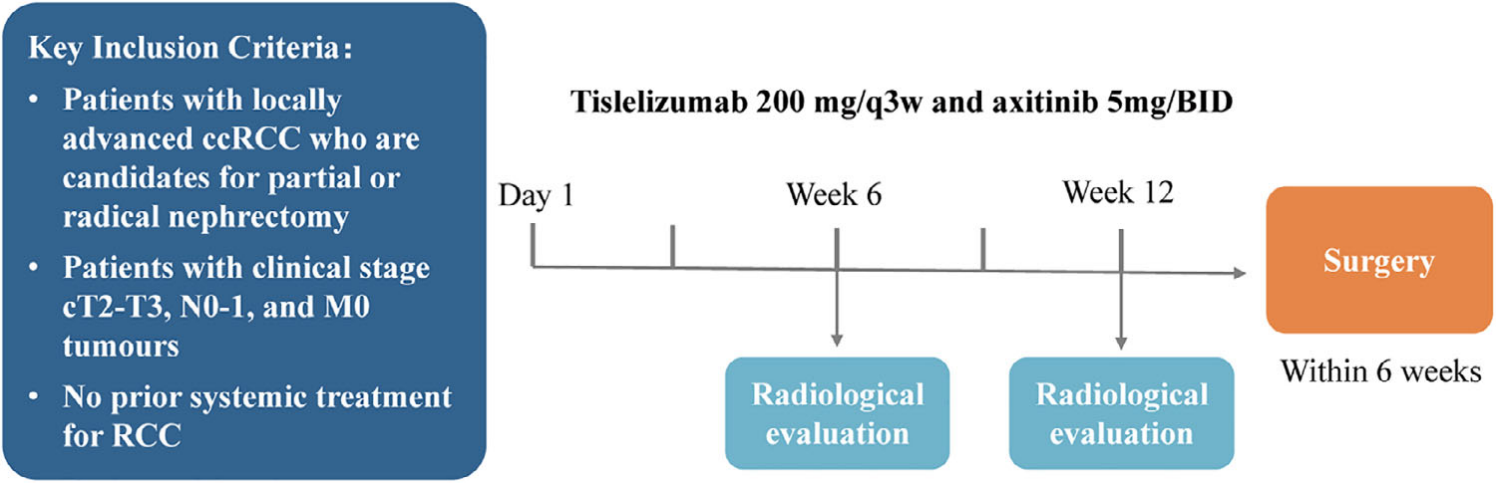
Experimental design.

First, patient eligibility was assessed and informed consent was obtained. Eligible patients then underwent a 12‐week combination treatment with axitinib and tislelizumab. The specific plan included oral administration of 5 mg axitinib twice daily for 12 weeks, alongside intravenous infusion of 200 mg tislelizumab on the first day of weeks 1, 4, 7, and 10. Each treatment cycle lasted 21 days, resulting in a total of four cycles over the 12‐week period. Following treatment, axitinib was discontinued for 7‒14 days. This pause was based on specific circumstances before proceeding to surgical treatment. After surgery, patients received 200 mg tislelizumab for immune maintenance therapy, depending on the pathological results or their health condition. It was administered intravenously every 3 weeks (Figure 4).

If a subject had severe toxic reactions during treatment, the dosage was adjusted. Following this adjustment, if the improvement was insufficient, we discontinued the treatment. After surgery, follow‐up was conducted once a month for the first 3 months, then every 3 months for 2 years, and every 6 months thereafter.

### 5.4 Endpoints and Evaluations

The ORR of tumors treated with medication before surgery served as the primary endpoint. In accordance with Response Evaluation Criteria in Solid Tumors V1.1, all subjects underwent renal CTA and CTU examinations at three distinct time points: 28 days prior to treatment, at the start of the third cycle of neoadjuvant therapy, and 7 days after conclusion of treatment (before surgery).

Response evaluation criteria in solid tumors: senior imaging experts assessed changes in tumor size and tumor thrombus length, calculating the PR and CR rates to derive the ORR.

The secondary endpoints include post‐operative DFS, overall survival (OS, reflects how long people live after they join a trial or start treatment), and safety outcomes. Throughout the treatment process, our team monitored and recorded patients’ adverse events in detail according to the Common Terminology Criteria for Adverse Events (V5.0).

### 5.5 Biomarker Analysis

We collected pre‐treatment biopsy samples, post‐treatment primary tumor samples, and matched paracancerous tissue samples from ccRCC patients. All samples were analysed using a targeted NGS panel of 437 cancer‐related genes (Geneseeq Prime, Nanjing Geneseeq Technologies Inc.) in a CLIA‐ and CAP‐certified clinical testing laboratory (Nanjing Geneseeq Technology Inc., Nanjing, China) (Supporting Information).

RNA‐seq data were obtained from the TCGA‐KIRC cohort (*N* = 510). Samples were filtered according to the presence of RTK/RAS pathway alterations. Immune cell infiltration was estimated using the ‘CIBERSORT’ algorithm [32].

### 5.6 Statistical Analysis

5.6

In this study, SPSS 25.0 was used for statistical analysis. The baseline characteristics of the participants, including age, gender, and clinical T‐stage, were analysed descriptively to calculate their mean, median, standard deviation, frequency, and percentage. Kaplan‒Meier curves were used to evaluate DFS and OS, and GraphPad Prism 10.1.2 was used for the statistical analysis of biomarkers.

Measurement data were summarized using the mean, standard deviation, median, upper and lower quartiles, and minimum and maximum values. Count data were summarized using frequencies and percentages. Group comparisons were conducted using methods appropriate to the data type: for quantitative data, the independent *t*‐test was applied when the assumptions of homogeneity of variance and normal distribution were met; otherwise, the Wilcoxon rank‐sum test was used. For categorical data, we utilized the chi‐square test, or the exact probability method if the chi‐square test was unsuitable, and the Wilcoxon rank‐sum test was employed for ranked data. All statistical tests, except for the main evaluation indicators, were conducted using two‐tailed tests, where *p* < 0.05 was considered statistically significant.

## Author Contributions

W.Y. and S.Z. contributed to conceptualization, methodology, investigation, formal analysis, and writing (original draft and revision). G.L. provided resources and supervision and participated in revision. H.L. and X.W. handled data curation and writing (review and editing). B.J. was responsible for visualization and writing (original draft). G.Z. focused on visualization and investigation. H.G. and C.J. (corresponding authors) supervised the project and contributed to revision. All the authors have read and approved the final manuscript.

## Funding

The study carried out was funded by General Program of National Natural Science Foundation of China (grant no. 82172777) (principal award recipient: Changwei Ji) and 2023 Jiangsu Postgraduate Research Innovation Plan (grant no. KYCX23_2101) (principal award recipient: Wenjin Yang).

## Ethics Statement

The study has been approved by the Ethics Committee of Drum Tower Hospital, affiliated with Nanjing University Medical School (approval number: 2021‐091‐03). It is also registered at clinicaltrials.gov in accordance with the Declaration of Helsinki (registration number: NCT05172440).

## Consent

All patients gave written informed consent. All authors have read and approved of its submission to this journal.

## Conflicts of Interest

The authors declare no potential conflicts of interests.

## Supporting information




**Table S1**. Specific gene variants.
**Table S2**. Representativeness of study participants.

## Data Availability

The data generated in this study are available within the article and its Supporting Information.
