# Supplementary material for: Tislelizumab Combined With Axitinib in Neoadjuvant Treatment of Locally Advanced Clear Cell Renal Cell Carcinoma: A Single‐Center, Phase II Clinical Study
Source: MedComm (2020). 2026 Feb 17;7(3):e70641. doi: 10.1002/mco2.70641 (PMC12914081; doi:10.1002/mco2.70641)
Supplement: Supplementary file 1 — Table S1. Specific gene variants. Table S2. Representativeness of study participants. [file MCO2-7-e70641-s001.docx]

Supplementary Text

Tislelizumab combined with axitinib in neoadjuvant treatment of locally advanced clear cell renal cell carcinoma: a single-center, phase II clinical study

Wenjin Yang^#1,2^, Shun Zhang^#1, 2^, Guangxiang Liu^1, 2^, Hao Li^3^, Xin Wang^1, 2^, Bo Jiang^1, 2^, Gutian Zhang^1, 2^, Hongqian Guo^*1,2,3^ ,Changwei Ji^*1,2,3^

^1^Department of Urology, Affiliated Drum Tower hospital, Medical School of Nanjing University, 321 Zhongshan Road, Nanjing, 210008, China;

^2^Institute of Urology, Nanjing University, 321 Zhongshan Road, Nanjing, 210008, China;

^3^Department of Urology, Nanjing Drum Tower Hospital Clinical College of Nanjing Medical University, 321 Zhongshan Road, Nanjing, 210008, China;

**#** Wenjin Yang and Shun Zhang are co-first authors of this article.

***Corresponding Authors**

Hongqian Guo, Department of Urology, Affiliated Drum Tower hospital, Medical School of Nanjing University, 321 Zhongshan Road, Nanjing, 210008, China Email: [dr.ghq@nju.edu.cn](mailto:dr.ghq@nju.edu.cn), Telephone: +86 13605171690;

Changwei Ji, Department of Urology, Affiliated Drum Tower hospital, Medical School of Nanjing University, 321 Zhongshan Road, Nanjing, 210008, China Email: jichangwei@nju.edu.cn, Telephone: +86 19822681999;

**Supplementary Text**

**DNA extraction and sequencing library construction**

Sample processing and sequencing analysis procedure were performed according to previously described methods [1,2]. Briefly, DNA was extracted using the QIAamp DNA FFPE Tissue Kit (QIAGEN, Dusseldorf, Germany) following the manufacturer’s instructions. Libraries preparations were performed using the KAPA Hyper Prep Kit (KAPA Biosystems, Wilmington, MA, USA) with optimized protocols. Libraries with different indices were pooled for targeted enrichment with GeneseeqPrime™ targeted NGS panel and xGen Lock-down Hybridization and Wash Reagents Kit (Integrated DNA Technologies), and then were sequenced on aNovaseq6000 platform (Illumina,).

**Mutation calling**

Trimmomatic was used for FASTQ file quality control [3]. Qualified data (QC above 15 and without extra N bases) was then mapped to human genome Hg19 using Burrows-Wheeler Aligner (BWA-mem, v0.7.12; <https://github.com/lh3/bwa/tree/master/bwakit>). Local realignment around indels and base quality score recalibration were performed using the Genome Analysis Toolkit (GATK 3.4.0; <https://software.broadinstitute.org/gatk/>) and duplicates were removed using Picard. VarScan2 was applied to detect single-nucleotide variations (SNVs) and INDELs. SNVs were filtered out if the mutant allele frequency (MAF) was less than 1% for tumor tissue. Copy number variations (CNVs) were called by FACETS (Fraction and Allele-Specific Copy Number Estimates from Tumor Sequencing) [4] to obtain tumor purity-, ploidy-, and clonal heterogeneity-adjusted copy number data. Gene fusions were identified by FACTERA [5]. TMB was counted by summing all base substitutions and indels in the coding region of targeted genes, including synonymous alterations to reduce sampling noise and excluding known driver mutations as they are over-represented in the Panel.

**Reference：**

1. Yang Z, Yang N, Ou Q, et al. Investigating Novel Resistance Mechanisms to Third-Generation EGFR Tyrosine Kinase Inhibitor Osimertinib in Non-Small Cell Lung Cancer Patients. *Clin. Cancer Res.: Off. J. Am. Assoc. Cancer Res.*, 2018;24 (13), 3097–3107.

2. Fang W, Ma Y, Yin J.C, et al. Comprehensive genomic profiling identifies novel genetic predictors of response to anti-PD-(L)1 therapies in non-small cell lung cancer. *Clin. Cancer Res.: Off. J. Am. Assoc. Cancer Res.*, 2019;25 (16), 5015–5026.

3. Bolger A.M., Lohse M., and Usadel B. Trimmomatic: A flexible trimmer for illumina sequence data. *Bioinform. (oxf. Engl.)*, 2014;30 (15), 2114–2120.

4. Shen R., and Seshan V.E. FACETS: Allele-specific copy number and clonal heterogeneity analysis tool for high-throughput DNA sequencing. *Nucleic Acids Res.*, 2016;44 (16), e131.

5. Newman A.M., Bratman S.V., Stehr H, et al. FACTERA: A practical method for the discovery of genomic rearrangements at breakpoint resolution. *Bioinform. (oxf. Engl.)*, 2014;30 (23), 3390–3393.

**Table S1**

specific gene variants

| **Gene** | **SD(N=7)** | **PR(N=4)** | **P-value** |
| --- | --- | --- | --- |
| **VHL** |  |  | 1.000 |
|  | 1(100.0%) | 0(0.0%) |  |
|  | 6(60.0%) | 4(40.0%) |  |
| **PBRM1** |  |  | 0.194 |
|  | 3(42.9%) | 4(57.1%) |  |
|  | 4(100.0%) | 0(0.0%) |  |
| **BAP1** |  |  | 0.491 |
|  | 6(75.0%) | 2(25.0%) |  |
|  | 1(33.3%) | 2(66.7%) |  |
| **MET** |  |  | 0.109 |
|  | 7(77.8%) | 2(22.2%) |  |
|  | 0(0.0%) | 2(100.0%) |  |
| **PTEN** |  |  | 0.491 |
|  | 5(55.6%) | 4(44.4%) |  |
|  | 2(100.0%) | 0(0.0%) |  |
| **ATM** |  |  | 1.000 |
|  | 6(66.7%) | 3(33.3%) |  |
|  | 1(50.0%) | 1(50.0%) |  |
| **FAT1** |  |  | 1.000 |
|  | 6(66.7%) | 3(33.3%) |  |
|  | 1(50.0%) | 1(50.0%) |  |

**Table S2**

Representativeness of Study Participants

| Cancer type | locally advanced clear cell renal cell carcinoma |
| --- | --- |
| Considerations related to: | |
| Sex | ccRCC is diagnosed more frequently in males than in females, with a male-to-female ratio of about 2:1. The incidence in males is significantly higher, reflecting a predominant male representation in clinical trials. |
| Age | The median age of participants in this study is 66 years, with an age range of 29 to 78 years. This aligns with the typical age distribution for ccRCC diagnoses, often seen in older adults. |
| Race/ethnicity | All participants in this study are Chinese patients. In China, the incidence of renal cell carcinoma, including ccRCC, predominantly affects the Han ethnic group, which constitutes the majority of the population. The inclusion of ethnic minorities is limited. |
| Geography | The study was conducted primarily in Jiangsu province and Anhui province in China, where the annual incidence of ccRCC is comparable to the national average of approximately 3-4 cases per 100,000 population. |
| Other considerations | Previous clinical trials of ccRCC have shown underrepresentation of ethnic minorities, which limits the understanding of treatment efficacy across diverse populations. This study aimed to focus specifically on the Chinese population. |
| Overall representativeness of this study | The age distribution of our study is similar to the average age at diagnosis for ccRCC in the literature. However, the study primarily includes male patients from the Han ethnic group, with limited diversity among participants. The overall representativeness is moderate. |
